# Supplementary material for: Deep Metric Learning for Scalable Gait-Based Person Re-Identification Using Force Platform Data
Source: Sensors (Basel). 2023 Mar 23;23(7):3392. doi: 10.3390/s23073392 (PMC10099366; doi:10.3390/s23073392)
Supplement: Supplementary file 1 [file sensors-23-03392-s001.zip › sensors-2257534-supplementary.pdf]

Supplementary Materials: Deep Metric Learning for Scalable Gait Based Person Re-identification Using Force Platform Data

1. Validation of performance evaluation method

An accessory experiment was conducted to validate the difficulty of the performance evaluation method implemented in this study. Namely, the FCNN architecture was implemented multiple times on the training, validation, and test sets from $D_{AS,AF}$ (with mini-batch size $N_X = 512$). Each time, the number of prior samples per identity (ID) available to compare with each query sample was altered in validation and test sets. The rest of the method was the same as detailed in Section 3.5 of the manuscript. The results are shown in Figure S6.

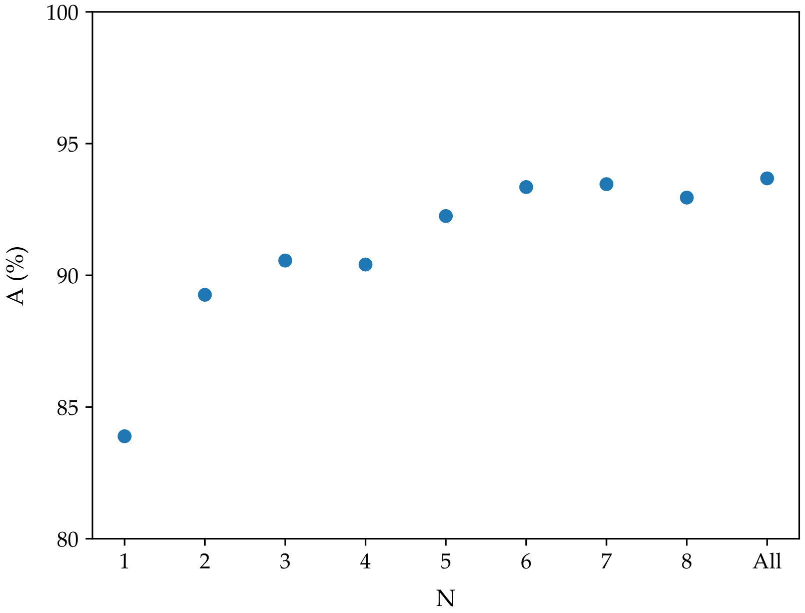

Figure S6. The relationship between the number of prior samples per identity (ID) available to compare with each query sample (N) and mean rank-1 accuracy (A) on test sets over seven-fold cross-validation using the fully-connected neural network architecture. The entire dataset ($D_{AS,AF}$) was used with training mini-batch size $N_X = 512$. In the 'All' condition, the common performance evaluation method was implemented, whereby *all* prior samples were available to compare with each query sample.

2. Comparison of mini-batch sizes

Across all models, mini-batch size $N_X = 512$ led to the highest mean F1 score on all four subsets, and the highest mean accuracy on all but $D_{PS,SF}$ (Tables S5 and S6). This batch size also led to superior convergence during training and validation (Figure S7).

Table S5. Accuracy (A) across all architectures according to mini-batch size N_X (provided as mean and min-max range over test sets with the best results in bold).

N_X	A %			
	$D_{AS,AF}$	$D_{AS,SF}$	$D_{PS,AF}$	$D_{PS,SF}$
32	78.08(68.17–84.78)	88.01(78.72–96.12)	78.10(66.81–90.79)	87.05(68.01–97.06)
64	81.64(71.39–88.91)	88.61(79.38–95.26)	81.79(75.22–88.82)	87.17(74.26–94.49)
128	82.20(75.6–90.57)	89.17(80.83–96.28)	82.26(73.49–88.89)	**89.60**(**77.57–97.43**)
256	82.62(76.17–90.31)	89.78(82.35–98.41)	83.38(73.92–92.43)	89.12(72.79–97.43)
512	**84.59**(**79.18–89.54**)	**90.35**(**81.84–97.88**)	**83.61**(**76.94–90.42**)	89.38(77.21–97.43)

Table S6. F1 score (F) across all architectures according to mini-batch size N_X (provided as mean and min-max range over test sets with the best results in bold).

N_X	F			
	$D_{AS,AF}$	$D_{AS,SF}$	$D_{PS,AF}$	$D_{PS,SF}$
32	0.77(0.68–0.84)	0.88(0.78–0.96)	0.76(0.62–0.91)	0.86(0.65–0.97)
64	0.81(0.70–0.88)	0.88(0.79–0.95)	0.80(0.72–0.88)	0.86(0.73–0.94)
128	0.81(0.75–0.90)	0.89(0.81–0.96)	0.81(0.72–0.89)	**0.89**(**0.77–0.97**)
256	0.82(0.75–0.90)	**0.90**(**0.82–0.98**)	**0.82**(**0.72–0.92**)	0.88(0.72–0.97)
512	**0.84**(**0.78–0.89**)	**0.90**(**0.82–0.98**)	**0.82**(**0.74–0.90**)	**0.89**(**0.77–0.97**)

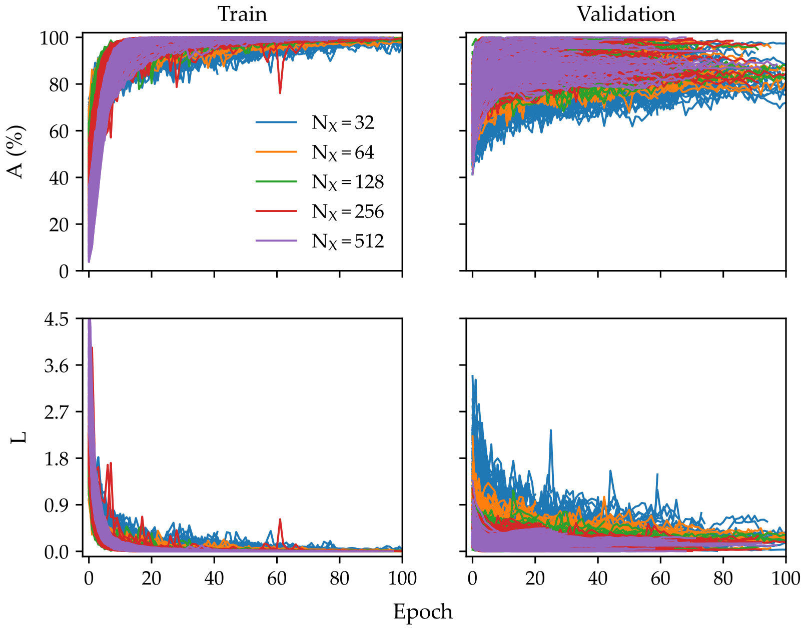

Figure S7. Accuracy (A) and loss (L) of all models over 100 epochs of training and validation with varying mini-batch size N_X. Convergence characteristics improved with increases in N_X. Using $N_X = 512$ led to the fastest and most consistent optimization, as well as the lowest variance between training and validation.

3. Most commonly mistaken individuals

See Table S7.

Table S7. Metadata of the 20 individuals who were responsible for 44 % of all incorrect predictions on $D_{AS,AF}$ (sorted by mean accuracy (A) across all test sets in ascending order). *Based on visual inspection of photographs Figure S8.

ID	A (%)	Age (y)	Sex	Mass (kg)	Height (m)	Session one shoe	Session two shoe	Same shoe category	Same shoe	Heel difference*
46	2.96	26	F	57.45	1.58	Women's ankle boot (heel)	Flat canvas (slip-on/laced)	N	N	Y
17	16.69	20	F	62.40	1.72	Women's ankle boot (heel)	Women's ankle boot (flat)	N	N	Y
120	24.06	33	F	86.45	1.73	Men's business shoe	Flip-flop	N	N	N
103	28.06	37	F	74.35	1.74	Athletic	Athletic	Y	N	-
113	30.83	35	F	77.25	1.61	Women's ankle boot (heel)	Ballet flat	N	N	Y
108	33.33	39	F	55.20	1.64	Women's ankle boot (heel)	Athletic	N	N	Y
39	35.06	31	F	54.45	1.56	Women's business shoe	Women's ankle boot (heel)	N	N	Y
52	44.20	23	M	64.90	1.83	Men's business shoe	Men's business shoe	Y	N	-
173	44.26	28	F	52.05	1.53	Women's ankle boot (flat)	Athletic	N	N	N
177	46.51	40	M	79.45	1.65	Athletic	Athletic	Y	Y	-
168	49.35	20	F	56.00	1.56	Women's business shoe	Ballet flat	N	N	Y
153	52.07	21	M	82.15	1.84	Men's business shoe	Flat canvas (slip-on/laced)	N	N	Y
179	52.07	41	M	70.50	1.70	Men's business shoe	Men's business shoe	Y	Y	-
192	53.14	24	F	63.60	1.72	Women's ankle boot (heel)	Flat canvas (slip-on/laced)	N	N	Y
146	56.69	44	F	95.60	1.74	Men's business shoe	Steel capped boot	N	N	N
181	60.47	25	F	70.20	1.70	Flat canvas (slip-on/laced)	Women's ankle boot (heel)	N	N	Y
20	63.80	21	F	68.75	1.84	Athletic	Women's ankle boot (heel)	N	N	Y
155	64.13	18	M	88.65	1.85	Flat canvas (slip-on/laced)	Flat canvas (slip-on/laced)	Y	Y	-
172	67.93	31	M	93.10	1.84	Athletic	Flat canvas (slip-on/laced)	N	N	N
167	73.25	18	M	67.95	1.68	Athletic	Men's business shoe	N	N	N

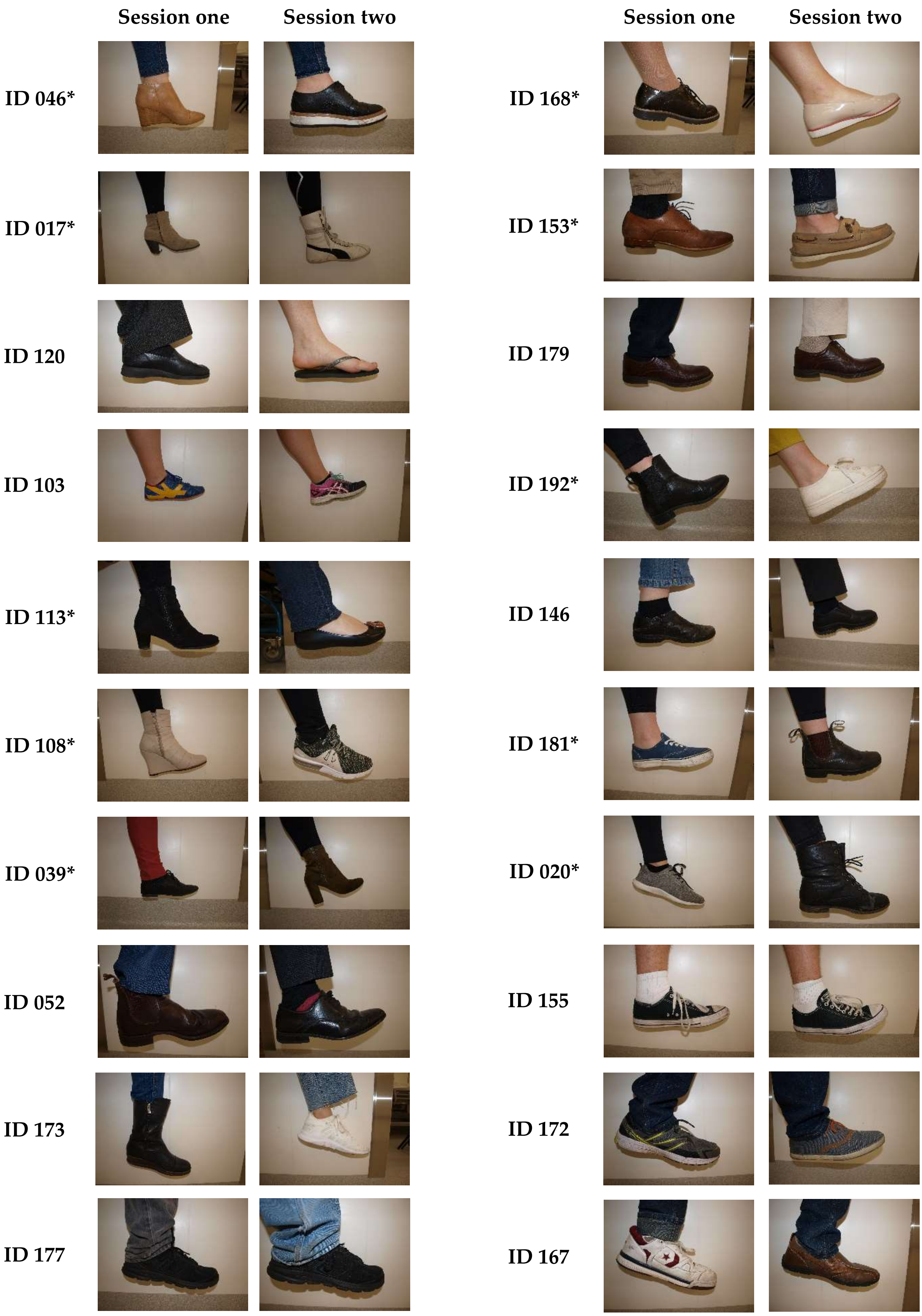

Figure S8. Photographs of footwear worn in each session by the 20 individuals who were responsible for 44% of all incorrect predictions on $D_{AS,AF}$ (sorted by mean rank-1 accuracy (A) on test sets in ascending order top-to-bottom, left-to-right). *classified as having difference in heel height based on visual inspection.
